# Tracing triggers of cardiac remodelling and heart failure

**DOI:** 10.1007/s12471-021-01597-0

**Published:** 2021-06-18

**Authors:** B. O. van Driel, E. E. Nollet

**Affiliations:** Department of Physiology, Amsterdam Cardiovascular Sciences, Amsterdam University Medical Centers, location VU University Medical Center, Vrije Universiteit Amsterdam, Amsterdam, The Netherlands

Predicting the disease course in patients with an inherited cardiomyopathy represents a significant contemporary problem in clinical practice. While it is well established that conditions such as familial hypertrophic cardiomyopathy (HCM) and familial dilated cardiomyopathy (DCM) are primarily of genetic aetiology, carriers of identical gene mutations show tremendous variation in terms of disease penetrance and severity. Therefore, it has been proposed that development of disease requires additional triggers, which may both elicit mutation-induced pathogenicity and impair cardiac function on top of the genetic burden [1].

Nationwide prospective cohort studies in Sweden have revealed that an elevated body mass index (BMI) early in life is a major risk factor for developing cardiomyopathy at a later age [2]. Compared with a normal BMI, obesity is associated with a three- and fivefold risk increase for developing HCM or DCM, respectively. A recent nationwide Korean study using population-based data demonstrated that metabolically unhealthy individuals (defined by presence of hypertension, hyperlipidaemia or diabetes mellitus) have a 1.5 times higher risk of developing HCM than metabolically healthy individuals [3]. Moreover, in patients with manifest HCM, obesity and related comorbidities (i.e. hypertension and diabetes mellitus type II) are associated with worse disease outcome [4], suggesting that triggers of cardiac remodelling and heart failure may be metabolic in nature and could therefore be measured to monitor or predict the disease course.

The heart is a metabolically flexible organ, which can consume nearly all types of energy substrates in order to generate ATP. It consumes an average of 30 g of fat and 20 g of carbohydrates per day. Based on substrate supply, or when confronted with abrupt disease or gradual disease progression, the heart may change its metabolism in an effort to more efficiently generate ATP. A shift in substrate metabolism from free fatty acids to glucose is an example of this metabolic flexibility, which has been observed to occur even before the onset of heart failure in mice with pressure-overloaded cardiac hypertrophy caused by aortic constriction [5].

Changes in cardiac metabolism can precede even more subtle changes on tissue level, such as hypertrophy or fibrosis, as shown by a reduction in myocardial energy efficiency in asymptomatic (genotype-positive, phenotype-negative) carriers of gene mutations associated with HCM [6]. Additionally, changes in cardiac metabolism can be measured by changes in circulating metabolites, as illustrated by a study that observed a decrease in circulating long-chain acylcarnitines in severe aortic stenosis patients within 24 hours after aortic valve replacement [7]. A recent study showed that metabolomic profiling of venous blood could aid in the diagnosis and timing of intervention for severe aortic stenosis [8].

Among the top 30 distinguishing metabolites between aortic stenosis patients and controls, 17 can be linked with nitric oxide metabolism. As nitric oxide mediates vasodilation, systemic changes therein may point to changes in vascular function. While the cardiomyocyte represents the main affected cell type in familial cardiomyopathies and in aortic stenosis-mediated remodelling of the heart, the notion that obesity represents a second disease hit indicates that other cell types may underlie disease progression. Tschöpe and Paulus proposed that a systemic pro-inflammatory state caused by comorbidities such as obesity and diabetes underlies endothelial dysfunction in heart failure patients with preserved ejection fraction (HFpEF) [9]. Results from the CHECK-HF registry, published in this issue of the Netherlands Heart Journal, show that comorbidities are highly prevalent in HFpEF [10].

In recent years, we have come to appreciate that several cardiomyopathies, including HCM, in fact present as an HFpEF-like syndrome, as HCM is also characterised by preserved ejection fraction and diastolic dysfunction. While the mutant protein is expressed in cardiomyocytes and not in vascular (endothelial) cells, reduced cardiac perfusion has been shown in HCM patients, in particular in patients with a muscle-specific pathogenic variant [11]. The observation of reduced coronary flow reserve in HCM patients with normal coronary angiograms led to the concept of microvascular (endothelial) dysfunction as a secondary pathomechanism in HCM development [12]. A recent study of samples from patients with obstructive HCM showed lower capillary density in females than in males [13], which coincided with more severe diastolic dysfunction. Moreover, capillary density was lower in end-stage failing hearts compared with samples taken from obstructive HCM, indicating that reduced capillary density underlies progression to heart failure in this patient group.

Thus far, research on the role of microvascular dysfunction and regression in disease onset and progression in inherited cardiomyopathy is mostly descriptive. Clearly, comprehensive studies on the role of obesity-related systemic changes and coincident endothelial dysfunction in the development of cardiomyopathy are warranted.
